# Interpreting measures of tuberculosis transmission: a case study on the Portuguese population

**DOI:** 10.1186/1471-2334-14-340

**Published:** 2014-06-18

**Authors:** Joao Sollari Lopes, Paula Rodrigues, Suani TR Pinho, Roberto FS Andrade, Raquel Duarte, M Gabriela M Gomes

**Affiliations:** 1 Instituto Gulbenkian de Ciência, Apartado 14, 2781-901 Oeiras, Portugal; 2 Faculdade de Ciências e Tecnologia, Universidade Nova de Lisboa, 2829-516 Caparica, Portugal; 3 Instituto de Física, Universidade Federal da Bahia, Campus Universitário de Ondina, 40210-340 Salvador, Brazil; 4 Centro Hospitalar de Vila Nova de Gaia/Espinho, 4434-502 Vila Nova de Gaia, Portugal; 5 Departamento de Epidemiologia Clínica, Medicina Preventiva e Saúde Pública, Faculdade de Medicina da Universidade do Porto, 4200-319 Porto, Portugal; 6 Instituto de Saúde Publica, Universidade do Porto, 4050-600 Porto, Portugal

**Keywords:** Heterogeneity, Sojourn times, Transmission dynamics, Tuberculosis epidemiology

## Abstract

**Background:**

Tuberculosis remains a high burden for Human society despite considerable investments in its control. Unique features in the history of infection and transmission dynamics of tuberculosis pose serious limitations on the direct interpretation of surveillance data and call for models that incorporate latent processes and simulate specific interventions.

**Methods:**

A transmission model was adjusted to the dataset of active tuberculosis cases reported in Portugal between 2002 and 2009. We estimated key transmission parameters from the data (i.e. time to diagnosis, treatment length, default proportion, proportion of pulmonary TB cases). Using the adjusted model to the Portuguese case, we estimated the total burden of tuberculosis in Portugal. We further performed sensitivity analysis to heterogeneities in susceptibility to infection and exposure intensity.

**Results:**

We calculated a mean time to diagnose of 2.81 months and treatment length of 8.80 months in Portugal. The proportion defaulting treatment was calculated as 0.04 and the proportion of pulmonary cases as 0.75. Using these values, we estimated a TB burden of 1.6 million infected persons, corresponding to more than 15% of the Portuguese population. We further described the sensitivity of these estimates to heterogeneity.

**Conclusions:**

We showed that the model reproduces well the observed dynamics of the Portuguese data, thus demonstrating its adequacy for devising control strategies for TB and predicting the effects of interventions.

## Background

Twenty years after considered to be a major global public health problem, tuberculosis (TB) remains a high burden for human society. In fact, WHO places TB as the second leading cause of death from an infectious disease, with almost two million deaths per year [1]. Moreover, according to this organization, one third of the world’s population is infected with TB and nearly 10 million people progress to an active state of the disease every year. Some aspects of TB transmission are slower than others in responding to changes in biologic, demographic, and socioeconomic conditions, resulting in a superposition of time scales that poses major challenges to the interpretation of transmission measures. A fraction of infected people progresses to active disease within two years of infection, while others may maintain a latent infection for decades with a reduced risk of disease progression. Complicating things further, those who have had the disease and have been successfully cured also maintain a risk of recurrent disease [2]. Furthermore, host susceptibility and rates of activation and reactivation of the disease appear to be related to co-morbidities (e.g. malnutrition, alcoholism, drug-addiction, diabetes, or HIV infection and other immunosuppressive diseases), high-risk of exposure to infection (e.g. health workers, prisoners), ageing and socioeconomic factors [3,4]. A challenge for mathematical modeling is to capture those aspects that are essential to the questions being addressed while maintaining a stylized structure that enables interpretation without compromising accuracy of the conclusions [5-8]. In this respect, the selection of the model is intimately connected to the posed question [7], but also to specificities of the settings being studied.

In this work we applied a mathematical model of TB transmission to Portuguese surveillance data. Recorded data on TB incidence in Portugal from ECDC [9] show a consistent decrease from 1999 to 2011 of 4.34% per year, resulting in a total decline in TB incidence of 52.13%. Nevertheless, Portugal registers the highest incidence among Western European countries (over 20/100,000, while a great majority is below 10/100,000), which reflects a considerable rate of TB transmission. Recent immigrants comprise only about 3% of overall yearly cases, and the 50% most common ages of TB patients vary vastly from 25 to 65 years old (note also that TB cases in children with less than 15 years old comprises an uncommonly significant proportion of almost 3%) [10]. Other common risk factors have some weight on the overall yearly TB cases in Portugal, for example being a prison inmate (about 2%), HIV-positive (about 14%) or drug-user (about 15%) [10]. Although not modeled explicitly, these factors are generally contemplated by our analysis of sensitivity to risk heterogeneity.

Initially, we applied a mathematical model for TB transmission under previously specified homogeneity assumptions [11]. For the analysis, we used data records from 2002 to 2009 provided by National Directorate of Health (Additional file 1). Using these data, we estimated parameter values of the model (i.e., detection time, treatment length, proportion of unsuccessful treatment, and proportion of pulmonary TB cases). Subsequently, we assessed the fit of the model by contrasting dynamic trajectories described by the parameterized model with real dynamics described by the data. The model was used to calculate the total burden of TB in Portugal considering the different stages of infection (i.e., primary infection, active infection not yet diagnosed, latent infection, and active infection under treatment). We further consider the existence of differential susceptibility to infection by applying an extended heterogeneous model based on previous work [8].

## Methods

### Analysis of treatment length and outcome in Portugal from 2002 to 2009

Surveillance data on TB for the period of 2002–2009 was provided by the Portuguese National Directorate of Health. These data, collected by medical practitioners, consisted of date of first symptoms, date of diagnosis and treatment starting, date of treatment ending, clinical form (i.e. pulmonary or extra-pulmonary), and treatment outcome [i.e. treatment completed (with or without laboratory confirmation); treatment failure; treatment default (i.e. lost to follow up); death (from tuberculosis or other causes)]. These data had been previously anonymized and, thus, no ethical approval was needed for the analysis presented here. From the dates of first symptoms and treatment starting and ending, we calculated time to detection and treatment length for each reported TB case - while the latter calculation is clearly objective, the former can raise some questions since retrospective self-report of symptoms is only a rough approximation for infection time. The proportion of treatment incompleteness was calculated by pooling together cases of default and treatment failure. The time to detection (1/*τ* yrs), treatment length (1/*δ*_*T*_ yrs), proportion of incomplete treatments (*ϕ*_*T*_) and proportion of pulmonary TB cases (*ν*) were estimated directly from the Portuguese dataset. To accommodate possible outliers from reporting/clerical errors or atypical cases, we discarded values of treatment length and detection time longer than 3 yrs. Following parameter estimations from raw data, cumulative data sets were created. Goodness-of-fit between the cumulative data and the theoretical expectation was assessed using two-sample Kolmogorov-Smirnov tests.

### Basic TB transmission model

In order to analyze the dynamics of TB epidemiology, we considered a TB transmission model modified from Gomes et al. [11]. In this model, susceptible individuals (class *S*) become primary infected with TB (class *P*) at rate *λ* yrs^−1^. From this class, a proportion *ϕ* enters an actively infected (and infectious) state (class *I*) at rate *δ* yrs^−1^, whereas the remaining individuals enter a latent infected state (class *L*) at the same rate. Actively infected individuals start TB treatment (class *T*) at rate *τκ* yrs^−1^, where *τ* is the inverse of the average time to detection and *κ* is the proportion of active TB cases that is actually detected and initiates treatment per year. Individuals under treatment are assumed to be neither infectious nor susceptible to reinfection. These individuals leave treatment at a rate *δ*_*T*_ yrs^−1^. A proportion *ϕ*_*T*_ leaves class *T* to *I* due to either treatment failure or default, while the remaining (1 - *ϕ*_*T*_) are successfully treated and transferred to class *L*. The model is described by the following system of ordinary differential equations:

(1) $$\begin{array}{l} \frac{\text{dS}}{\text{dt}}=\mu -\left(\lambda +\mu \right)S\\ \frac{\text{dP}}{\text{dt}}=\mathrm{\lambda S}+\mathrm{\sigma \lambda L}-\left(\delta +\mu \right)P\\ \frac{\text{dI}}{\text{dt}}=\phi \delta P+\mathrm{\omega L}+\phi_{T}\delta_{T}T-\left(\tau \kappa +\mu \right)I\\ \frac{\text{dL}}{\text{dt}}=\left(1-\phi \right)\delta P+\left(1-\phi_{T}\right)\delta_{T}T-\left(\sigma \lambda +\omega +\mu \right)L\\ \frac{\text{dT}}{\text{dt}}=\mathrm{\tau \kappa I}-\left(\delta_{T}+\mu \right)T \end{array}$$

The model parameters along with their typical values used herein are listed in Table 1. The force of infection is simply *λ* = *βνI*, where *β* is the transmission coefficient and *ν* is the proportion of pulmonary TB cases. Birth and death rates are assumed equal, fixed as *μ* = 1/80 yrs^−1^. The reinfection factor *σ* is the decreased chance of contriving TB by exogenous reinfection after previous exposure (either after active disease followed by successful treatment or after having only remained in a latent state). For this factor we considered *σ* = 0.5, as estimated in Gomes et al. [8] using a world-wide dataset. For the endogenous reactivation rate *ω*, we adopted *ω* = 0.0003 yrs^−1^, as estimated by Vynnycky et al. [12,13] for the population of the United Kingdom.

**Table 1:** Parameters of tuberculosis transmission models

**Symbol**	**Definition**	**Value**
*β*	Transmission coefficient	variable (yrs^−1^)
*ν*	Proportion of pulmonary TB cases	0.75
*μ*	Death and Birth rate	1/80 yrs^−1^
*δ*	Rate at which individuals leave *P* compartment	2 yrs^−1^
*ϕ*	Fraction of infected population developing active TB	0.05
*σ*	Reinfection factor	0.5
*ω*	Rate of endogenous reactivation	0.0003 yrs^−1^
*τ*	Inverse of time to detection	4.26 yrs^−1^
*κ*	Proportion of detected cases in a year	0.87
*δ*_*T*_	Inverse of treatment length	1.36 yrs^−1^
*ϕ*_*T*_	Fraction of treatment default and failure	0.04
*α*^a^	Low-risk factor	0.15 or variable
*γ*^a^	Proportion of low-risk group	0.98 or variable

^a^- Parameter used only in the heterogeneous model.

**Figure 1:**
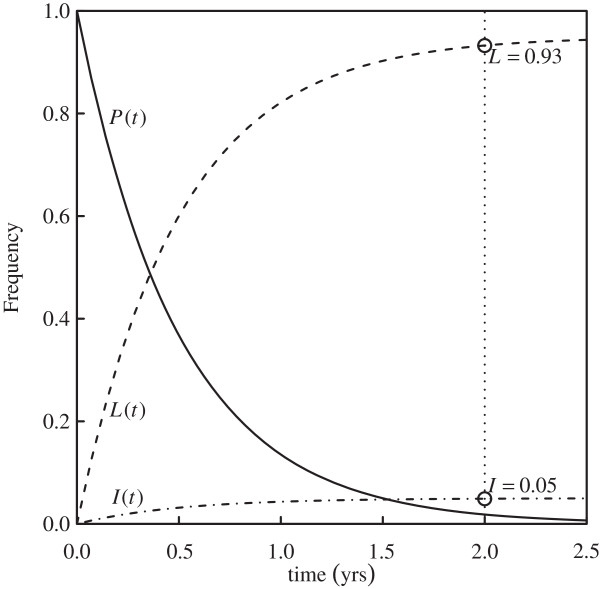
**Sojourn time distribution in the primary infection state.** Exponential distribution for the time infected individuals remain in primary state (*P*), and proportional progression to active disease (*I*) and latent (*L*) states. The curves are given by *P*(*t*) = *e*^− *δ* ⋅ *t*^, *I*(*t*) = *ϕ* ⋅ (1 − *e*^− *δ* ⋅ *t*^), *L*(*t*) = (1 − *ϕ*) ⋅ (1 − *e*^− *δ* ⋅ *t*^), with parameter values *ϕ* = 0.05 and *δ* = 2 yrs^−1^, in agreement with the expectation that 5% of primary cases progress to active disease within 2 years of infection (dotted-line).

Parameters describing the transition between the primary infection class *P* to *L* and *I* were revised. Numerous TB models do not consider a primary infection class *P* explicitly (see [7] and references therein). In our model, this simplification would correspond to setting the rate of progression out of *P* as *δ* → ∞. Instead, we specified a primary infection stage based on information from the literature. After invasion of TB bacteria, some balance between the host immune system and the pathogen occurs. In about 5% of cases, the infection progresses to an active state within 2 years, and an additional 5% become actively infected later on [2,14]. Hence, we specified a rate (*δ*) out of the primary stage, *P*, and a proportion (*ϕ*) to represent those progressing directly from primary infection to active disease. Assuming that 5% of the individuals become actively infected within 2 years of exposure, we set parameter values compatible with observed rates, as *ϕ* = 0.05 and *δ* = 2 yrs^−1^ (see Figure 1). Treatment class is described by four parameters, specifically rate of detection (*τ*), proportion of detected cases (*κ*), treatment length (1/*δ*_*T*_) and the proportion of incomplete treatment (*ϕ*_*T*_). Parameter *κ* was taken from WHO estimates for Portugal as 87% [1]. The remaining parameters were estimated directly from the Portuguese dataset as stated before.

The basic reproduction number *R*_0_ was obtained using the approach in [15]:

(2) $$R_{0}=\frac{\beta \nu \delta \left(\delta_{T}+\mu \right)\left(\phi \mu +\omega \right)}{\mu \left(\delta +\mu \right)\left[\left(\mu +\omega \right)\left(\tau \kappa +\delta_{T}+\mu \right)+\delta_{T}\tau \kappa \left(1-\phi_{T}\right)\right]}$$

System (1) has two equilibria: a disease-free equilibrium *E*_1_ = (1,0,0,0,0) stable when *R*_0_ < 1; and an endemic equilibrium *E*_2_ = (S^*^,P^*^,I^*^,L^*^,T^*^), where all coordinates are nonzero, stable when *R*_0_ > 1. Details on the model and its equilibrium points are in Additional file 2.

### TB model assuming heterogeneous infection risk

The mathematical model represented by system (1) can be extended as in [8] to enable a heterogeneous risk of infection in the population. In this implementation the population is composed of a subpopulation 1 with low risk of infection, where the force of infection is affected by a factor *α*_1_ < 1, and a subpopulation 2 with high risk of infection, where the force of infection is increased by a factor *α*_2_ > 1. Parameters *γ*_1_ and *γ*_2_ are the proportions of the population in each group, such that *γ*_1_ + *γ*_2_ = 1. The average risk factor is normalized by considering *γ*_1_*α*_1_ + *γ*_2_*α*_2_ = 1, thus, heterogeneity is fully parameterized by *α*_1_ (≡α) and *γ*_1_ (≡γ). The model is expressed as

(3) $$\begin{array}{l} \frac{dS_{i}}{\text{dt}}=\gamma_{i}\mu -\left(\lambda_{i}+\mu \right)S_{i}\\ \frac{dP_{i}}{\text{dt}}=\lambda_{i}S_{i}+\sigma \lambda_{i}L_{i}-\left(\delta +\mu \right)P_{i}\\ \frac{dI_{i}}{\text{dt}}=\phi \delta P_{i}+\omega L_{i}+\phi_{T}\delta_{T}T_{i}-\left(\tau \kappa +\mu \right)I_{i}\\ \frac{dL_{i}}{\text{dt}}=\left(1-\phi \right)\delta P_{i}+\left(1-\phi_{T}\right)\delta_{T}T_{i}-\left(\sigma \lambda_{i}+\omega +\mu \right)L_{i}\\ \frac{dT_{i}}{\text{dt}}=\tau \kappa I_{i}-\left(\delta_{T}+\mu \right)T_{i} \end{array}$$

where *λ*_*i*_ = *α*_*i*_*βνI*, *i* = {1,2} and *α* and *γ* can take values from the interval (0,1). For reference, [8] estimated *α* = 0.15 and *γ* = 0.98 using a world-wide dataset.

The basic reproduction number $R_{0}^{'}$ for system (3) is also expressed by equation (2). This can be derived by noting that in the heterogeneous model the average number of infections contributed by a single infectious individual is the sum $R_{0}^{'}=\gamma_{1}\alpha_{1}R_{0}+\gamma_{2}\alpha_{2}R_{0}$, where the first and second terms account for transmission to the low- and high-risk groups, respectively. Considering that *γ*_1_ + *γ*_2_ = 1 and *γ*_1_*α*_1_ + *γ*_2_*α*_2_ = 1, and that *γ*_1_ ≡ *γ* and *α*_1_ ≡ *α*, we obtain $R_{0}^{'}=\gamma \alpha R_{0}+\left(1-\alpha \gamma \right)R_{0}$, or simply $R_{0}^{'}=R_{0}$. From now on the basic reproduction number is represented by *R*_0_ irrespective of whether we refer to the homogeneous or heterogeneous model.

System (3) has also two equilibria, a disease-free equilibrium $E_{1}^{'}$ = (*γ*,1 - *γ*,0,0,0,0,0,0,0,0) stable when *R*_0_ < 1, and an endemic equilibrium $E_{2}^{'}$ = ($S_{1}^{*}$, $S_{2}^{*}$, $P_{1}^{*}$, $P_{2}^{*}$, $I_{1}^{*}$, $I_{2}^{*}$, $L_{1}^{*}$, $L_{2}^{*}$, $T_{1}^{*}$, $T_{2}^{*}$), whose coordinates are all nonzero for the considered range of parameter values, which is stable when *R*_0_ > 1.

## Results

### Parameter estimation from TB data in Portugal

**Figure 2:**
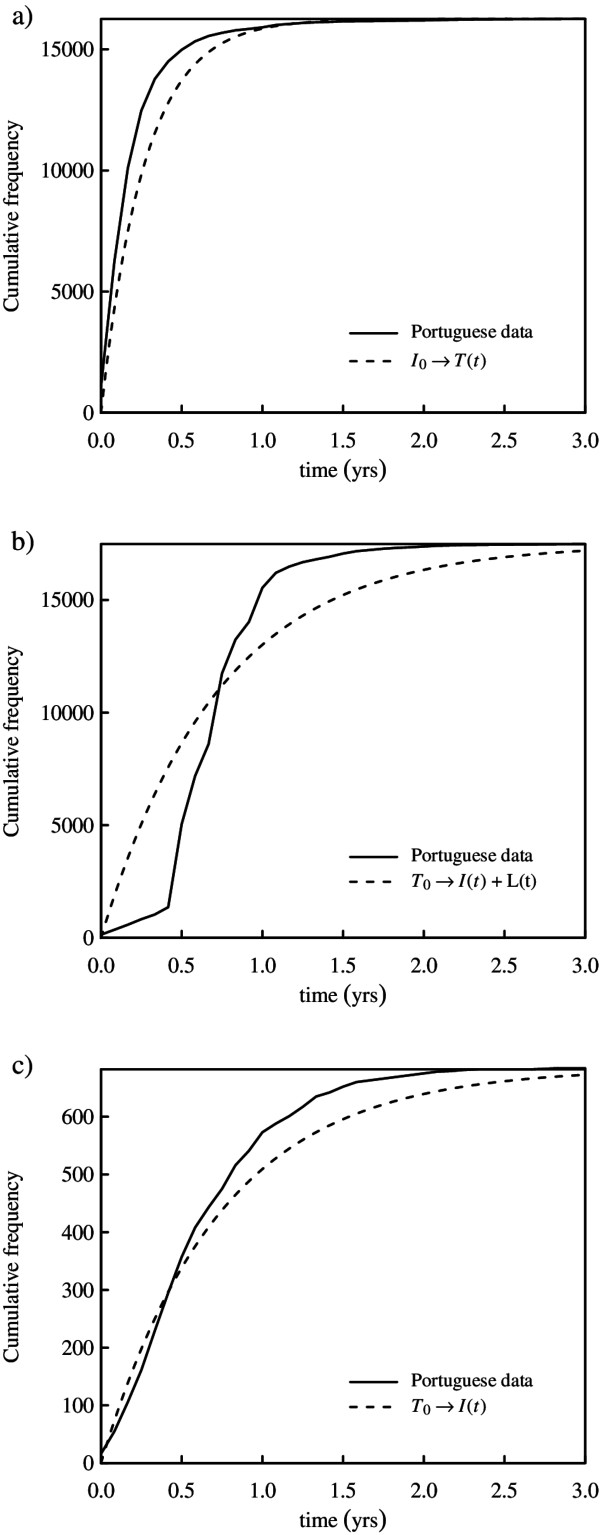
**Superposition of observed data and theoretical expectations. a)** Cumulative frequency of detection of tuberculosis infected patients and theoretical expectations for the inflow to class *T* assuming *τ* = 4.26 yrs^−1^. **b)** Cumulative frequency of TB treatment length and theoretical expectations for the outflow from class *T* assuming *δ*_*T*_ = 1.36 yrs^−1^. **c)** Cumulative frequency of TB treatment defaulters and theoretical expectations for the flow from class *T* to class *I* assuming *δ*_*T*_ = 1.36 yrs^−1^ and *ϕ*_*T*_ = 0.04.

For the analysis on detection time, we selected 16,257 cases (where detection time ≤ 3 yrs) corresponding to 99.7% of all data. The calculated mean value of detection time and its standard error was 2.81 ± 0.02 months (*τ* = 4.26 yrs^−1^), while its median was 2 with an interquartile-range also of 2. Given these parameters, the model expectation for the flow into the under-treatment class *T* of a cohort *I*_0_ is

(4) $$I_{0}\to T\left(t\right):=I_{0}\cdot \left(1-e^{-\tau \kappa \cdot t}\right)$$

where *I*_0_ = 16,257, *τ* = 4.26 yrs^−1^ and *κ* = 0.87. Figure 2a shows a superposition of expression (4) and the cumulative frequency of detection times of the selected cases. The agreement between the exponential distribution and the observed sojourn times in the infectious class *I* is impressive given that only the central tendency of the raw data was fitted (D = 0.243, p-value = 0.226).

For the analysis of treatment length we selected 17,477 cases (where treatment length ≤ 3 yrs) corresponding to 99.9% of all data. The calculated mean value of treatment length and its standard error was 8.80 ± 0.03 months (*δ*_*T*_ = 1.36 yrs^−1^), while its median was 9 with an interquartile-range of 4. As before, the model expectation for the flow out of the under-treatment class *T* of a cohort *T*_0_ becomes

(5) $$T_{0}\to I\left(t\right)+L\left(t\right):=T_{0}\cdot \left(1-e^{-\delta_{T}\cdot t}\right)$$

where *T*_0_ = 17,477. Figure 2b shows a superposition of expression (5) with *δ*_*T*_ = 1.36 yrs^−1^, and the cumulative frequency of treatment length of the selected cases. These results also show some agreement between the observations and the exponential curve (D = 0.4595, p-value = 6.746 × 10^−4^).

As for the ratio of treatment incompleteness (i.e. treatment default or failure), we considered the data from 2002–2009 with known outcome. The ratio of defaults and failures to the total number of treatments was calculated as 0.04. Note also that, in the case of the Portuguese dataset, the rate of failures is insignificant compared to the rate of defaults (Table 2), thus, *ϕ*_*T*_ can be approximated as the rate of defaults in TB treatment outcomes. Considering the flow out of *T*_0_ to class *I* only, the theoretical reconstruction can be written as

**Table 2:** Outcome of tuberculosis treatment in Portugal, 2002–2009

**Outcome**	**2002**	**2003**	**2004**	**2005**	**2006**	**2007**	**2008**	**2009**
Death	126	126	145	142	129	130	122	100
Default	110	93	92	98	102	61	65	46
Failure	1	6	3	3	4	4	0	0
Completed	2,109	2,074	2,162	2,091	2,146	2,045	1,971	1,198
Unassigned	44	39	50	26	42	91	125	865

(6) $$T_{0}\to I\left(t\right):=T_{0}\cdot \left(1-e^{-\delta_{T}\cdot t}\right)\cdot \varphi_{T}$$

where *T*_0_ = 17,477. Figure 2c shows the superposition of expression (6), where *δ*_*T*_ = 1.36 yrs^−1^ and *ϕ*_*T*_ = 0.04, and the cumulative frequency of treatment incompleteness for the selected cases. Again, there is an impressive agreement between the observations and the theoretical curve, showing that sojourn times in treatment *T* are also approximately exponential (D = 0.3226, p-value = 0.079. Ties on observed data were discarded for a correct calculation of the p-value).

Finally, for the proportion of pulmonary TB cases, we considered once again the full data from 2002–2009 (Table 3). The ratio of pulmonary TB cases was 0.75.

**Table 3:** Clinical form under tuberculosis treatment in Portugal, 2002–2009

**Clinical form**	**2002**	**2003**	**2004**	**2005**	**2006**	**2007**	**2008**	**2009**
Pulmonary	1,748	1,717	1,844	1,752	1,850	1,749	1,745	1,643
Extra-pulmonary	643	621	610	608	573	582	538	566

### Epidemiology of TB in Portugal

ECDC recorded data show a consistent decline from 1999 to 2011 on TB incidence in Portugal [9], suggesting a non-steady state of TB transmission. Nevertheless, as an initial approximation, this study assumes equilibrium conditions for TB transmission, with an average number of reported TB cases of 2348.25 cases per year (Table 2). According to the Portuguese National Institute of Statistics, the population size of Portugal during this period was approximately 10.56 million people, resulting in a rate of 22.24 treatments initiated per 100,000 person-years.

From system (1) and considering that TB transmission is at equilibrium, TB incidence can be calculated as

(7) $$Y=\varphi \delta P+\mathrm{\omega L}+\varphi_{T}\delta_{T}T=\left(\tau \kappa +\mu \right)I$$

considering that *τκ* > > *μ*, we can simplify expression (7) as *Y* = *τκI*. Thus, TB incidence in Portugal can be simply calculated as the rate of entering treatment *Y* = 2.224 × 10^−4^ per person-years (i.e. 22.24 per 100,000 person-years), while the prevalence is approximated by I^*^ = *Y*/*τκ* = 4.261 × 10^−5^ (i.e. 4.261 per 100,000 persons). Assuming that system (1) is at equilibrium and considering the parameter values of Table 1 and that I^*^ = 4.261 × 10^−5^, we calculated the value of *β* and of the proportion of individuals in the remaining classes of the model (Table 4). Thus, we obtained *R*_0_ = 1.116 (*β* = 72.358 yrs^−1^). These estimates resulted in expectations for T^*^ that are congruent with the data collected in Portugal (see Additional file 2). Sensitivity analysis to *ω*, *σ* and *δ* showed the robustness of these estimates (Additional file 3). Note that given the uncertainties regarding TB reinfection, we considered a range of values for sensitivity analyses for *σ* that encompass the possibility that a previous TB infection increases risk of getting reinfected [16].

**Table 4:** Estimates of active disease prevalence and basic reproduction number for tuberculosis transmission models (1) and (3)

**System**^**a**^	**S**	**P (10**^**−3**^**)**	**I (10**^**−5**^**)**	**L**	**T (10**^**−4**^**)**	***R***_**0**_
1	0.844	1.059	4.261^b^	0.155	1.148	1.116
3	0.930	1.316	4.261^b^	0.069	1.148	2.237
3 with varying *α* and *γ*	pr: 0.844	pr: 1.059	4.261^b^	pr: 0.059	1.148^c^	pr: 1.116
	Pr: 0.940	Pr: 1.348		Pr: 0.155		Pr: 2.421

*Abbreviations:* S Susceptible, P Primary infection, I Active Infection, L Latent Infection, *R*_0_ Reproduction number, pr 0.5% percentile, Pr 99.5% percentile.

^a^- parameter values according to Table 1 unless stated otherwise.

^b^- estimated directly from tuberculosis incidence.

^c^- estimation is not a function of *α* or *γ*.

Considering the Portuguese population size, the estimated values correspond approximately to 11,000 people with primary TB infection, 1,600,000 with latent infection, 1,200 patients under treatment and 500 undiagnosed actively infected persons.

### Effect of considering a heterogeneous model

Following the same reasoning as used for system (1), we assumed for system (3) that TB transmission in Portugal from 2002 to 2009 is close to equilibrium and that the fraction of actively TB infected individuals ($I_{1}^{*}$ + $I_{2}^{*}$) was 4.261 × 10^−5^. By considering the parameter values of Table 1, we obtained estimates of the stationary values (Table 4) and *R*_0_ = 2.237 (*β* = 145.024 yrs^−1^).

These estimates were obtained assuming risk heterogeneity governed by parameters *α* and *γ* previously estimated from global tuberculosis data [8]. Additionally, we analyzed the sensitivity of estimated proportions of individuals in each class and of *R*_0_ estimation to variation in these parameters (Additional file 3 and Table 4). To avoid very low (i.e., close to 0) and very high (i.e., close to 1) values of *α* and *γ*, which greatly affect estimations, we consider values between 0.005 and 0.995. This sensitivity analysis results in particularly large variations in susceptible and latently infected classes (Table 4). Considering the Portuguese population size, the estimated values indicate approximately 11,000 to 14,000 people with primary TB infection, 600,000 to 1,600,000 with latent infection, 1,200 patients under treatment and 500 undiagnosed actively infected persons. *R*_0_ varies from 1.116 to 2.421 (*β* varies from 72.358 to 156.98 yrs^−1^).

### Trends of TB prevalence with transmission coefficient and *R*_0_

**Figure 3:**
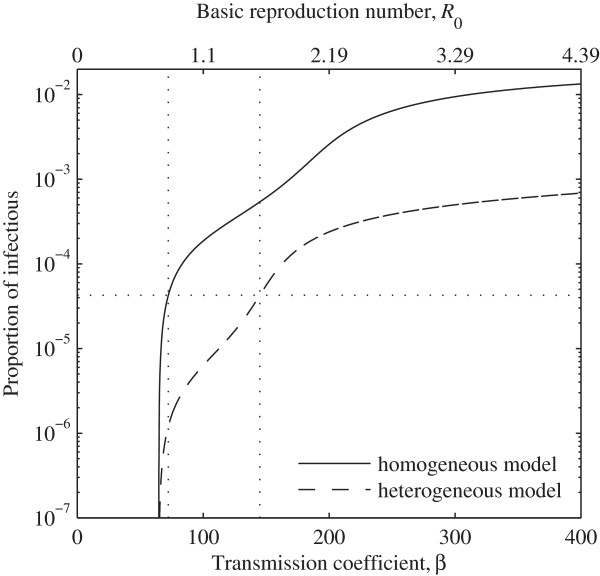
**Prevalence of active TB as a function of transmission parameters.** Proportion of infectious individuals as a function of *β* and *R*_0_. Curves represent the endemic equilibria according to the homogeneous system (1) (full line) and the heterogeneous system (3) (dashed line), using parameter values specified in Table 1. Estimates of *β* and *R*_0_ for the same proportion of infectious individuals under both models are marked (dotted lines).

Assuming that systems (1) and (3) are at equilibrium, we can calculate how the proportion of infected individuals (I^*^ for system (1) and $I_{1}^{*}$ + $I_{2}^{*}$ for system (3)) vary with transmission coefficient *β* (Additional file 2). Figure 3 represents the proportion of infectious individuals in system (1) (full line) and system (3) (dashed-line) for different values of *β* and *R*_0_. The curves were obtained by considering the parameter values in Table 1 with the proportion *γ* and the susceptibility factor *α* of the low-risk group fixed to the values estimated in [8]. The dotted-lines indicate the values of *β* and *R*_0_ for systems (1) and (3) corresponding to the estimated proportion of infectious individuals 4.261 × 10^−5^.

## Discussion

With a TB prevalence of over 20 cases per 100,000 individuals, Portugal is one of the countries with the highest TB burden in the European Union (along with Bulgaria, Estonia, Latvia, Lithuania, Poland and Romania) [9]. Furthermore, there are worries that the current economic crisis may increase TB burden in this country due to a consequent increase of unemployment, wealth inequality and general deterioration of the living conditions [17,18]. For this reason, developing a mathematical model that suits the Portuguese scenario is important as to better understand the dynamics of this transmission system, identify key parameters in the control of TB and to inform policies by predicting the impact of interventions.

Despite the clear need for a better understanding of TB in Portugal, this work is the first that considers the complexity of TB transmission to estimate the Portuguese TB burden. Its main limitations are: the calculation of TB detection time from retrospective self-diagnosed data; the simplifications of the model, particularly, not assuming age-structure nor emigration; and the assumption of a steady-state for TB transmission. As such, although the results provide a valuable indication of TB burden in each stage of TB infection, they should be interpreted with caution.

In the present work, we had access to Portuguese clinical and socio-demographic data and calculated directly the standard detection time for TB as of about 2.81 months and the typical treatment length as 8.80 months. These values are comfortably within the range recommended by WHO [1]. The default proportion in Portugal was calculated as 4%, a value that is somewhat lower than previously reported (6 to 30%) [19]. Using these calculated values along with parameters taken from the literature, we assessed our proposed model against the Portuguese data by comparing expected temporal dynamics with those recorded in the database (Figure 2). Agreement is impressive given the simple and unconstrained nature of the model, indicating its adequacy to the study of TB transmission in Portugal. For this reason, although more complex heterogeneous models have been proposed [20,21], we are confident on the suitableness of the considered model.

The burden of TB in a country is difficult to measure due to the different stages of the disease and the impossibility to quantify directly from surveillance data the proportion of individuals in some of these stages. In the adopted model we considered four stages of TB infection: primary infection; active disease not yet diagnosed; active disease undergoing treatment; and latent infection. Surveillance data is limited to patients entering the treatment stage. However, by considering a specific mathematical model we were able to estimate the total burden of TB in Portugal as of about 1.6 million infected individuals (i.e., about 11,000 with primary TB infection, 1,600,000 with latent infection, 1,200 under-treatment and about 500 undiagnosed active infections), corresponding to an *R*_0_ of 1.116. This value for *R*_0_ is very much in line with previous studies [22,23].

Nonetheless, we recognize that, as evidenced by Murphy et al. [24] and Gomes et al. [8], individual variation in susceptibility to infection may change dramatically the transmission dynamics of TB leading to significant differences in the interpretation of population measures. Murphy and co-authors consider genetic factors as a partial cause for heterogeneity, but, as previously noted, there is much uncertainty associated to these factors [25]. Another possible cause for heterogeneity is whether individuals are vaccinated or not, however, given the inconsistent protection conferred by the Bacillus Calmette–Guérin vaccine, there is also considerable uncertainty related to the impact of vaccination [26]. Bacaer and co-authors [27] considered a heterogeneous model to study jointly TB and HIV epidemiology. This model divided the population in high and low risk of TB infection, depending if the persons were HIV positive or negative, and was developed to analyze data from South Africa, a country where about 12% of the population is infected with HIV [28]. Dowdy et al. [29] considered a heterogeneous transmission model to study TB data from Rio de Janeiro. In this study the high and low risk division was done by considering geographically distributed prevalence data, however, the existence of clearly defined “hotspots” of TB cases in this setting is mostly due to slumbers and other well-defined regions with a high impact of risk factors and social determinants. We argue, then, that heterogeneity in TB infection risk in Portugal is likely to result from a differential impact of biological and social determinants to different sections of the population [3,17]. An often neglected caveat of introducing heterogeneity in TB transmission models is that the factors that drive heterogeneity may break assumptions of random mixing (e.g. being in prison or nursing-home, working on health-care, living in slumbers). In Portugal, however, the factors that have been implied with heterogeneity in TB risk are not markedly associated with closed-groups (e.g. HIV and drug-use [9], unemployment, wealth inequality and living conditions [17]). Given the difficulty to categorize the Portuguese population as having a high or low risk of infection so that the world-wide estimations from [8] could be directly verified and refined, we calculated the TB burden from a spectrum of profiles of heterogeneous susceptibility. Hence, we estimated a range of possible TB burden of between 0.6 and 1.6 million infected individuals (i.e., 11,000 to 14,000 with a primary TB infection, 600,000 to 1,600,000 latent-infected, and the same 1,200 under-treatment and 500 undiagnosed actively-infected as verified in the homogeneous scenario), corresponding to *R*_0_ from 1.116 to more than 2.421. The wide range of values for TB burden is mostly attributed to the wide range of estimates of the proportion of latently-infected individuals. Considering an increasing trend on the reactivation rate of latent infections [13], it is of great importance to characterize the heterogeneity of a population and, thus, to estimate with accuracy the proportion of latently-infected individuals in a population.

In the comparison between homogeneous and heterogeneous models, although we considered the same proportion of infectious individuals (i.e. 4.261 × 10^-5^), we estimated a possible range of values for *R*_0_ and *β* in the heterogeneous case that can be considerable distinct from the ones estimated in the homogeneous case. This interesting dynamic is better explored in Figure 3, where we calculated the proportion of infected individuals for various values of *β* and *R*_0_ while considering systems 1 and 3 (with fixed values of *α* and *γ*). These results show that TB estimates of transmission coefficient *β* (and of *R*_0_) may vary greatly depending on the model considered. Examining the effect of heterogeneity in *R*_0_ is especially important since, as noted before, its use in TB may be problematic if all the dynamics of the transmission system are not considered [30,31].

## Conclusions

We proposed a mathematical model for TB transmission, which fits well the dynamics described by records of the Portuguese National Directorate of Health, as a tool for intervention impact studies. Under homogeneity assumptions, we estimated an upper bound for the total burden of TB infection in Portugal of 1.6 million individuals (more than 15% of the Portuguese population). Accounting for heterogeneities in susceptibility and exposure to infection led to lower estimates of infection prevalence.

## Competing interests

The authors declare that they have no competing interests.

## Authors’ contributions

JSL and MGMG conceived the study; JSL executed the study and wrote first draft; STRP, RFSA, PR and MGMG conceived the models; RD provided data access and interpretation; all authors discussed results and wrote the paper. All authors read and approved the final manuscript.

## Pre-publication history

The pre-publication history for this paper can be accessed here:

http://www.biomedcentral.com/1471-2334/14/340/prepub

## Supplementary Material

Additional file 1 Portuguese TB dataset. Click here for file

Additional file 2 Basic models and their equilibrium points. Click here for file

Additional file 3 Sensitivity analyses. Click here for file
