# Supplementary material for: Interpreting measures of tuberculosis transmission: a case study on the Portuguese population
Source: BMC Infect Dis. 2014 Jun 18;14:340. doi: 10.1186/1471-2334-14-340 (PMC4069091; doi:10.1186/1471-2334-14-340)
Supplement: Additional file 1 — Portuguese TB dataset. [file 1471-2334-14-340-S1.doc]

### Additional file 1 – Portuguese TB dataset.

The Portuguese dataset maintained by the National Health System consists of clinical and socio-demographic data collected between 2002 and 2009 from patients diagnosed with TB in public hospitals in four major Portuguese regions (North, Center, Lisbon and Tagus Valley, and South). These data include the time of first symptoms, times of starting and ending of treatment, treatment outcome and clinical form of the disease (i.e. pulmonary or extra-pulmonary). From these data we calculated the number of individuals entering treatment per year while breaking those numbers per treatment outcome (Figure S1a) and per clinical form (Figure S1b).

**Figure S1. Frequency of tuberculosis treatment cases.**

**a)** Frequency of tuberculosis treatment outcomes per year. **b)** Frequency of tuberculosis clinical form per year. Color codes for the outcomes are: white - patient death; light grey - treatment default; grey - treatment failure; dark grey - treatment completed; black - under treatment. Color codes for the clinical form are: grey - pulmonary form; dark grey - extra-pulmonary form.
