# Supplementary material for: Interpreting measures of tuberculosis transmission: a case study on the Portuguese population
Source: BMC Infect Dis. 2014 Jun 18;14:340. doi: 10.1186/1471-2334-14-340 (PMC4069091; doi:10.1186/1471-2334-14-340)
Supplement: Additional file 2 — Basic models and their equilibrium points. [file 1471-2334-14-340-S2.doc]

### Additional file 2 – Basic models and their equilibrium points.

*Interpretation of R_0_*

The expression for *R*_0_ for system 1 given by equation 2,

$R_{0}=\frac{\beta\nu\delta\left(\delta_{T}+\mu\right)\left(\varphi\mu+\omega\right)}{\mu\left(\delta+\mu\right)\left[\left(\mu+\omega\right)\left(\tau\kappa+\delta_{T}+\mu\right)+\delta_{T}\tau\kappa\left(1-\varphi_{T}\right)\right]}$,

can be written as the sum of different progressions into active TB. In the simpler case *δ_T_* = 0 (i.e., treatment for TB gives total immunity), we obtain equation A.1 of Gomes et al. [1]

$R_{0}^{*}=\frac{\beta\nu}{\left(\tau\kappa+\mu\right)}\left[\frac{\varphi\delta}{\left(\delta+\mu\right)}+\frac{\left(1-\varphi\right)\delta}{\left(\delta+\mu\right)}\frac{\omega}{\left(\omega+\mu\right)}\right]$, (1)

where the first factor gives the average number of secondary cases produced by a single individual with active TB in a completely susceptible population during its infectious period. Following the same reasoning as in the work of Gomes and co-authors [1], the first and second terms in the second factor account for progression for active TB through either class *P* and class *L*, respectively. When *δ_T_* > 0, treated individuals have an extra opportunity to progress to active TB without being re-exposed to an infected individual, either by defaulting treatment or progressing through class *L*. This extra contribution for the infectious period is expressed by the term

$$\frac{\left(\tau\kappa+\mu\right)\left(\delta_{T}+\mu\right)\left(\omega+\mu\right)}{\mu\left[\left(\mu+\omega\right)\left(\tau\kappa+\delta_{T}+\mu\right)+\delta_{T}\tau\kappa\left(1-\varphi_{T}\right)\right]}=1+\frac{\tau\kappa}{\left(\tau\kappa+\mu\right)}\left(\frac{\varphi_{T}\delta_{T}}{\left(\delta_{T}+\mu\right)}+\frac{\left(1-\varphi\right)}{\left(\delta_{T}+\mu\right)}\frac{\omega}{\omega+\mu}\right)+$$

$+\left[\frac{\tau\kappa}{\left(\tau\kappa+\mu\right)}\left(\frac{\varphi_{T}\delta_{T}}{\left(\delta_{T}+\mu\right)}+\frac{\left(1-\varphi\right)}{\left(\delta_{T}+\mu\right)}\frac{\omega}{\omega+\mu}\right)\right]^{2}+...$, (2)

that results from the countless opportunities the infected individual has to repeat this event. The full expression for *R*_0_ is the product of equations (1) and (2).

*Equilibrium points of systems 1 and 3*

System (1) has two equilibria. The first one is known as the trivial equilibrium and corresponds to a disease free equilibrium, *E*_1_ = (1,0,0,0,0). The second one corresponds to an endemic equilibrium, *E*_2_ = (*S*^*^,*P*^*^,*I*^*^,*L*^*^,*T*^*^), where all coordinates are nonzero. The values for *E*_2_ coordinates are expressed in terms of the roots of a second order polynomial, where only the positive real root is relevant. Considering the values of the parameters expressed in Table 1, we can write the coordinates as a function of *β*, such as

$\begin{matrix} S^{*}=A \\ P^{*}=\left(c_{1}+c_{2}\beta+c_{3}\beta\cdot A\right)/\beta\\ I^{*}=\left(d_{1}+d_{2}\beta+d_{3}\beta\cdot A\right)/\beta\\ L^{*}=\left(e_{1}+e_{2}\beta+e_{3}\beta\cdot A\right)/\beta\\ T^{*}=\left(f_{1}+f_{2}\beta+f_{3}\beta\cdot A\right)/\beta\end{matrix}$, (S1)

where *A* takes the value of the positive root of

$$a_{1}\beta\cdot x^{2}+\left(a_{2}\beta+a_{3}\right)\cdot x+a_{4}=0$$

and *a_p_*, *p* = {1, 2, 3, 4}, and *c_q_*, *d_q_*, *e_q_*, *f_q_*, *q* = {1, 2, 3} are real numbers.

System (3) has also two equilibria. The trivial equilibrium $E_{1}^{'}$ = (*γ*,1 - *γ*,0,0,0,0,0,0,0,0) and an endemic equilibrium $E_{2}^{'}$ = ($S_{1}^{*}$,$S_{2}^{*}$,$P_{1}^{*}$,$P_{2}^{*}$,$I_{1}^{*}$,$I_{2}^{*}$,$L_{1}^{*}$,$L_{2}^{*}$,$T_{1}^{*}$,$T_{2}^{*}$), where, for the range of parameters considered, all coordinates are nonzero and are expressed in terms of the roots of a fourth order polynomial. Again, the only relevant set of coordinates of $E_{2}^{'}$ comes from the positive real root. Considering the parameter values in Table 1, we can also write the coordinates of the endemic equilibrium of system (3) such as

$\begin{matrix} S_{1}^{*}=B \\ S_{2}^{*}=\left[g_{1}\beta\cdot B^{3}+\left(g_{2}\beta+g_{3}\right)\cdot B^{2}+\left(g_{4}\beta+g_{5}\right)\cdot B+g_{6}\right]/\beta\\ P_{1}^{*}=\left[h_{1}\beta\cdot B^{3}+\left(h_{2}\beta+h_{3}\right)\cdot B^{2}+\left(h_{4}\beta+h_{5}\right)\cdot B+h_{6}\beta+h_{7}\right]/\beta\\ P_{2}^{*}=\left[i_{1}\beta\cdot B^{3}+\left(i_{2}\beta+i_{3}\right)\cdot B^{2}+\left(i_{4}\beta+i_{5}\right)\cdot B+i_{6}\beta+i_{7}\right]/\beta\\ I_{1}^{*}=\left[j_{1}\beta\cdot B^{3}+\left(j_{2}\beta+j_{3}\right)\cdot B^{2}+\left(j_{4}\beta+j_{5}\right)\cdot B+j_{6}\beta+j_{7}\right]/\beta\\ I_{2}^{*}=\left[k_{1}\beta\cdot B^{3}+\left(k_{2}\beta+k_{3}\right)\cdot B^{2}+\left(k_{4}\beta+k_{5}\right)\cdot B+k_{6}\beta+k_{7}\right]/\beta\\ L_{2}^{*}=\left[l_{1}\beta\cdot B^{3}+\left(l_{2}\beta+l_{3}\right)\cdot B^{2}+\left(l_{4}\beta+l_{5}\right)\cdot B+l_{6}\right]/\beta\\ L_{2}^{*}=\left[m_{1}\beta\cdot B^{3}+\left(m_{2}\beta+m_{3}\right)\cdot B^{2}+\left(m_{4}\beta+m_{5}\right)\cdot B+m_{6}\right]/\beta\\ T_{1}^{*}=\left[n_{1}\beta\cdot B^{3}+\left(n_{2}\beta+n_{3}\right)\cdot B^{2}+\left(n_{4}\beta+n_{5}\right)\cdot B+n_{6}\beta+n_{7}\right]/\beta\\ T_{2}^{*}=\left[o_{1}\beta\cdot B^{3}+\left(o_{2}\beta+o_{3}\right)\cdot B^{2}+\left(o_{4}\beta+o_{5}\right)\cdot B+o_{6}\beta+o_{7}\right]/\beta\end{matrix}$, (S2)

where *B* takes the value of the positive root of

$$b_{1}\beta\cdot x^{4}+\left(b_{2}\beta+b_{3}\right)\cdot x^{3}+\left(b_{4}\beta+b_{5}\right)\cdot x^{2}+\left(b_{6}\beta+b_{7}\right)\cdot x+b_{8}=0$$

and *g_r_*, *l_r_*, *m_r_*, *r* = {1, 2, 3, 4, 5, 6} and *h_s_*, *i_s_*, *j_s_*, *k_s_*, *o_s_*, *s* = {1, 2, 3, 4, 5, 6, 7} are real numbers.

*Estimating stationary values for T from data*

From the observed TB incidence in Portugal *Y* = 2.224×10^-4^, according to equation (7) we calculated the stationary value of the proportion of infections *I*^*^ = 4.261×10^-5^. Using this estimate and considering the parameter values of Table 1, we calculated *T*^*^ = 1.148×10^-4^. The stationary value for patients under treatment, *T*^*^, can also be calculated directly from the Portuguese data. Considering only the cases under treatment from 2003 to 2008 in order to avoid missing data, we calculated the total number of patients under treatment at any given day (Figure S2). These data showed a very stable behavior with a mean of 1728.61 cases. Considering the Portuguese population of 10.56 million people, we obtained an estimate of 1212.03 patients under treatment, which is close to the one obtained using TB incidence data.

**Figure S2. Observed cases under treatment in Portugal at any given time (in days) during 2003-2008.**

**References**

1. Gomes MGM, Rodrigues P, Hilker FM, Mantilla-Beniers NB, Muehlen M, Paulo AC, Medley GF: **Implications of partial immunity on the prospects for tuberculosis control by post-exposure interventions.** *J Theor Biol* 2007, 248: 608–617.
