# Supplementary material for: Interpreting measures of tuberculosis transmission: a case study on the Portuguese population
Source: BMC Infect Dis. 2014 Jun 18;14:340. doi: 10.1186/1471-2334-14-340 (PMC4069091; doi:10.1186/1471-2334-14-340)
Supplement: Additional file 3 — Sensitivity analyses. [file 1471-2334-14-340-S3.doc]

### Additional file 3 – Sensitivity analyses.

In the analysis of the Portuguese dataset, we assume fixed values for the model parameters as listed in Table 1. For the parameters *μ* and *κ* (characteristic of the Portuguese population) and parameter *φ* (related to TB intra-host dynamics), we are confident in the values used. For the reinfection factor *σ*, the endogenous reactivation rate *ω* and rate *δ* of leaving class *P* we are less certain about their values. Thus, we perform sensitivity analyses for each parameter separately while considering the general TB transmission model (Figure S3 and Table S1) and fixing *β* to the estimated value. These analyses show that the TB model is reasonably robust to different values of *σ*, *ω* and *δ*.

For the heterogeneous model, we considered two more parameters, which control the level of heterogeneity in susceptibility of the Portuguese population: proportion of low-risk *γ* and low-risk factor *α*. Obtaining specific estimates for these two parameters is out of the scope of this study, hence, we estimate the proportion of individuals in each class and of the basic reproduction number for a range of values of *γ* and *α*. Since very low (i.e., close to 0) and very high (i.e., close to 1) values of *α *and *γ* greatly affect estimations, we restrict the analysis to values between 0.005 and 0.995 (Figure S4 and Table S2). These results show that the stationary values for classes *S*_1_ and *S*_2_ and *L*_1_ and *L*_2_, as well as the *R*_0_, are particularly sensitive to these parameters.

**Figure S3. Sensitivity analyses to endogenous reactivation *ω*, reinfection factor *σ* and rate *δ* for leaving class *P*.**

**a)** Sensitivity of the proportion of individuals in classes *S* (double-line), *P* (full-line), *L* (dashed-line), *I* (dashed and dotted -line) and *T* (dotted-line) to the rate of endogenous reactivation, *ω*. **b)** Sensitivity of *R*_0_ to the rate of endogenous reactivation, *ω*. **c)** Sensitivity of the proportion of individuals in classes *S* (double-line), *P* (full-line), *L* (dashed-line), *I* (dashed and dotted -line) and *T* (dotted-line) to the reinfection factor, *σ*. **d)** Sensitivity of *R*_0_ to the reinfection factor, *σ*. **e)** Sensitivity of the proportion of individuals in classes *S* (double-line), *P* (full-line), *L* (dashed-line), *I* (dashed and dotted -line) and *T* (dotted-line) to the rate for leaving class *P*, *δ*. **b)** Sensitivity of *R*_0_ to the rate for leaving class *P*, *δ*.

**Figure S4. Sensitivity analysis to proportion of low-risk *γ* and low-risk factor *α*.**

**a)** Sensitivity of proportion of individuals in classes *S*_1_ and *S*_2_. **b)** Sensitivity of proportion of individuals in classes *P*_1_ and *P*_2_. **c)** Sensitivity of proportion of individuals in classes *L*_1_ and *L*_2_. **d)** Sensitivity of reproduction number *R*_0_.

**Table S1. Sensitivity analysis to rate of Endogenous reactivation *ω* and reinfection factor *σ* for system (1)**.

| Symbol | Range | $S^{*}$ | $P^{*}$($10^{-3}$) | $L^{*}$ | $T^{*}$ ($10^{-4}$) | $R_{0}$ |
| --- | --- | --- | --- | --- | --- | --- |
| *ω* | [0, 0.001] | m: 0.784  M: 0.907 | m: 0.605  M: 1.523 | m: 0.092  M: 0.214 | 1.148^a^ | m: 1.081  M: 1.124 |
| *σ* | [0, 1.25] | m: 0.834  M: 0.855 | m: 1.030  M: 1.092 | m: 0.144  M: 0.165 | 1.148^a^ | m: 1.025  M: $\infty$ |
| *δ* | [1, 4] | m: 0.843  M: 0.844 | m: 0.529  M: 2.118 | m: 0.155  M: 0.155 | 1.148^a^ | m: 1.115  M: 1.117 |

Abbreviations: m, minimum value; M, maximum value.

^a^ - estimate is not a function of the parameter.

**Table S2. Sensitivity analysis to proportion *γ* and factor *α* describing the low-risk group for system (3)**.

| Symbol | Range | $S_{1}^{'*}+S_{2}^{'*}$ | $P_{1}^{'*}+P_{2}^{'*}$ | $L_{1}^{'*}+L_{2}^{'*}$ | $T_{1}^{'*}+T_{2}^{'*}$ | $R_{0}^{'}$ |
| --- | --- | --- | --- | --- | --- | --- |
| *α* and *γ* | (0, 1) | m: 0.844  pr: 0.844  Pr: 0.940  M: 0.996 | m: $1.06\times10^{-3}$ pr: $1.06\times10^{-3}$ Pr: $1.35\times10^{-3}$ M: $1.52\times10^{-3}$ | m: 0.002  pr: 0.059  Pr: 0.155  M: 0.155 | $1.15\times10^{-4}$^a^ | m: 1.116  pr: 1.116  Pr: 2.421  M: $\infty$ |

Abbreviations: m, minimum value; M, maximum value; pr, 0.5% percentile; Pr, 99.5% percentile.

^a^ - estimate is not a function of the parameters.
